# Spatial Analysis of Tuberculosis Cases in Migrants and Permanent Residents, Beijing, 2000–2006

**DOI:** 10.3201/1409.071543

**Published:** 2008-09

**Authors:** Zhong-Wei Jia, Xiao-Wei Jia, Yun-Xi Liu, Christopher Dye, Feng Chen, Chang-Sheng Chen, Wen-Yi Zhang, Xiao-Wen Li, Wu-Chun Cao, He-Liang Liu

**Affiliations:** Capital Medical University, Beijing, People’s Republic of China (PRC) (Z.-W. Jia); State Key Laboratory of Pathogen and Biosecurity, Beijing (Z.-W. Jia, W.-Y. Zhang, W.-C. Cao); Chinese Center for Disease Control and Prevention, Beijing (X.W. Jia); Chinese PLA General Hospital, Beijing (Y.-X. Liu); World Health Organization, Geneva, Switzerland (C. Dye); Nanjing Medical University, Nanjing, PRC (F. Chen); Fourth Military Medical University, Xi’an, PRC (C.-S. Chen, H.-L. Liu); Chinese Academy of Sciences, Beijing (X.-W. Li)

**Keywords:** pulmonary tuberculosis, migrant population, spatial analysis, Extra Poisson model, research

## Abstract

To determine the role of the migrant population in the transmission of tuberculosis (TB), we investigated the distribution and magnitude of TB in permanent residents and migrant populations of Beijing, People’s Republic of China, from 2000 through 2006. An exploratory spatial data analysis was applied to detect the “hot spots” of TB among the 2 populations. Results, using the data obtained from 2004–2006, showed that people who migrated from the western, middle, and eastern zones of China had a significantly higher risk of having TB than did permanent residents. These findings indicate that population fluctuations have affected the rate of TB prevalence in Beijing, and interventions to control TB should include the migrant population.

## Abstract 2

Population fluctuation is a risk factor for TB in Beijing.

Tuberculosis (TB) is a reemerging infectious disease and a substantial public health problem in metropolitan Beijing, People’s Republic of China. The case notification rate and mortality rate of TB have ranked third and first, respectively, among rates for 37 notifiable infectious diseases since 2000 (*1*–*5*). The proportion of cases in migrants is increasing year by year. In 2006, the migrant population accounted for 1,638 of 4,088 cases, a proportion that is 80% of the total number of cases of the permanent residents. Prevention and control of TB among the migrant population are now great challenges in Beijing (*1*–*3*).

“Migrant population” is a characteristic concept in China, resulting from the *hukou* system. *Hukou* is a household registration system in which a permanent residency permit for 1 place is issued by the government to each family. Every family has a *hukou* booklet that records information about the family members, including name, birth date, relationship with each other, marriage status (and to whom if married), employer, and residence address. Everyone has a *hukou* in China, which is assigned to a baby at birth, according to the residence of his or her parents. To move *hukou* from 1 place to another is usually difficult (*6*).

Before 1980, *hukou* was extremely important for citizens of China. They were required to stay at the small area where their *hukou* was until they died. They could travel, but, if they did, they had no access to jobs, public services, education, or even food. It was just like visiting other countries with a B-1 (business) visa. After 1980, many circumstances changed with the reform of the Chinese economy. In practice, *hukou* does not play as important role as before, which has led many farmers to leave their homeland and go to cities to seek jobs. A migrant population has thus arisen.

For this migrant population to be managed, migrants are required to register for a temporary residence permit (TRP) in the Department of Migrant Population Management of the local public security bureau if they are >16 years of age and stay in a place for >1 month (*7*). Employers are responsible for registering TRP for their workers in the district where the work place is located. For persons without a job, the community they reside in has to supervise their registration to obtain a TRP. The period of validity for TRP is usually 1 year. If the migrant changes his or her job or residence before the TRP expires, the person must register a new TRP in the new location and cancel the old one at the same time (*7*).

By law, migrants have the same rights as permanent residents. However, in terms of economic status, many differences exist between migrants and permanent residents. For example, the migrant population is restricted in access to public health and welfare services in the host areas because the local financial allocation is mainly based on the number of registered *hukou,* and the local governments only provide for the persons with *hukou*. Additionally, because migrants are usually employed at a lower income, they are unlikely to be covered by social medical insurance and thus are unlikely to seek healthcare promptly when they are ill. All of these factors indicate that the migrant population usually holds a lower economic status in host areas.

Economic development and urbanization in China have increased in recent years. In 2001, Beijing had ≈2 million migrants and 11 million permanent residents (*8*). By the end of 2006, the migrant population exceeded the 5 million mark and accounted for one third of the total population in Beijing (*9*). Moreover, most migrants come from the rural areas, where prevalence of TB is high (*10*). They travel between their hometowns and metropolitan Beijing and, consequently, bring the disease to Beijing.

Previous studies indicated that the reemergence of TB seemed to be associated with the mass migrant population (*10*–*14*). However, detailed assessments of the potential effects of the migrant population are hampered by limited information about the reported cases from this population. In the current study, we analyzed the spatial distribution of the patients with diagnosed TB cases in both migrants and permanent residents using geographic information system (GIS) techniques (*15*). We also attempted to identify the “hot spot” areas in the 2 populations. Finally, GIS-based multilevel extra Poisson regression models analysis was conducted to clarify the impact of the migrant population on the reemergence and transmission of TB in Beijing (*16*).

## Materials and Methods

### Data Collection and Management

**Figure 1:**
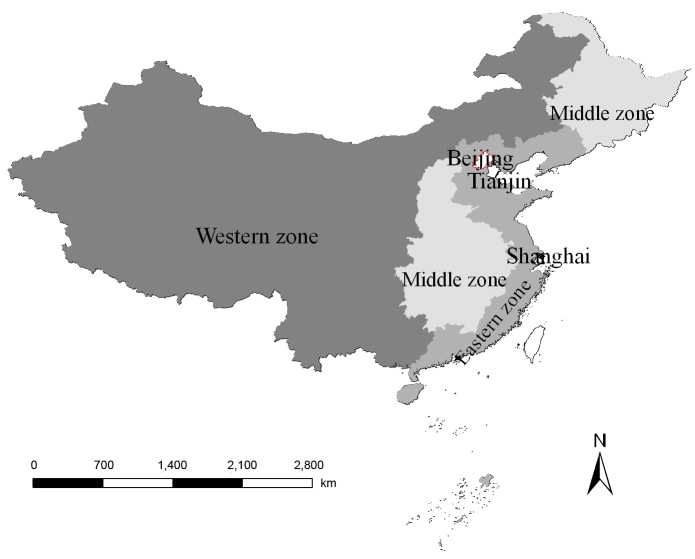
The 4 zones of China. These divisions were made on the basis of economic and geographic factors.

The data on all the TB cases reported in Beijing from 2000 through 2006 were obtained from the Beijing Institute for Tuberculosis Control, which specializes in TB prevention and research and is responsible for supervision of TB control in 18 districts of metropolitan Beijing. The cases that met the diagnostic criteria of TB issued by Ministry of Public Health in 2003 (*17*) were included in the analyses. The data include information on age, origin, current address, and date of TB onset. To assess the contribution of the migrant population from different areas, the case origins were divided into 4 zones, according to economic status and geography, i.e., western zone (including Shanxi, Gansu, Qinghai, Ningxia, Inner Mongolia, Xinjiang, Tibet, Sichuan, Chongqing, Guizhou, Guangxi, Yunnan Provinces, or other administrative regions), middle zone (including Heilongjiang, Jilin, Shanxi, Henan, Anhui, Hubei, Hunan, Jiangxi Provinces), eastern zone (including Liaoning, Hebei, Shandong, Jiangsu, Zhejiang, Fujian, Guangdong, Hainan Provinces), and 2 municipalities (Tianjin and Shanghai) (Figure 1). The zonal classification corresponded to that of the Report on Nationwide Survey on Epidemiology of Tuberculosis in 2000 (*18*) and thus was easily used for comparison. The case data have been stratified by age, gender, origin, and onset date of TB; age was divided into 3 groups: 1) 0–14 years, 2) 15–64 years, and 3) >65 years. All the TB cases were coded according to the address where they resided (geo-coded) and matched to a 1:100,000 digital map of Beijing by using ArcGIS version 9.1 software (ESRI Inc., Redlands, CA, USA).

**Figure 2:**
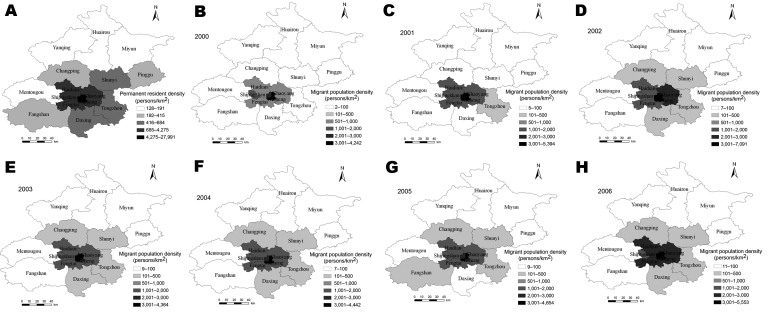
Population density of permanent residents and migrant population in Beijing, 2000–2006. Because the population density of permanent residents showed little change from 2000 through 2006, we showed the mean density of permanent residents in panel A. Panels B through H denote the density of migrant population from 2000 through 2006, respectively.

The demographic data of permanent residents and migrant population for each district were obtained from the 2000–2006 censuses, provided by Beijing Municipal Public Security Bureau (*8*,*9*,*19*–*23*). The 18 districts of Beijing, covering a total surface area of ≈16,800 km^2^, had 11,976,900 permanent residents and 5,475,000 migrants in 2006 (*9*). On the basis of these data, the population densities of each district in different years were calculated and displayed on the digital map of Beijing (Figure 2).

The permanent residents in our study were defined as those who reside in Beijing with registered *hukou* in Beijing, and the migrant population was defined as those who had been residing in Beijing >1 month but whose *hukou* were still held in their homelands. Persons originally from other countries were beyond the scope of our current study. A shape file of property boundary data of 18 districts in Beijing 2003 obtained from the Ministry of Water Resources of the People’s Republic of China was used to generate visual presentations with 1:100,000 scale by using ArcGIS 9.1.

### Statistical Analysis

**Figure 3:**
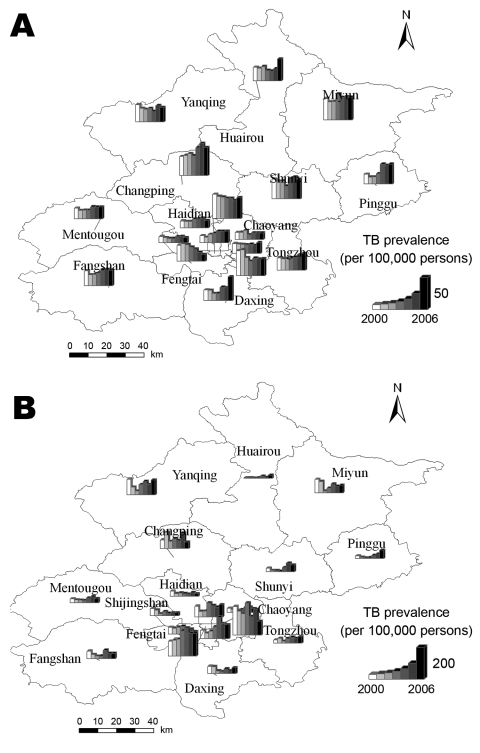
The prevalence rate of tuberculosis (TB) among the permanent residents and migrant population in Beijing, 2000–2006. The district graph unit consists of 7 bars, which denote the prevalence rate of TB from 2000 through 2006, respectively. A) Change in TB prevalence among permanent residents. B) Change in TB prevalence among migrant population.

The dynamic changes in population densities and the TB case notification rate of both migrant population and permanent residents from 2000 to 2006 were displayed by district on the digital map of the Beijing municipality (Figures 2, 3). Global Moran’s *I* statistics with z score test and Getis’s G_i_^*^ statistics, which specify 10 km as the threshold of distance, have been used to detect the spatial distribution and the hot spots of TB in the 2 populations (*24*,*25*). Global Moran’s *I* is used to discern spatial autocorrelation of TB cases in the study area and disclose the spatial pattern of disease with z score at the district level. A statistically significant (z score >1.96) estimate of *I* indicates that neighboring districts (within 10 km) have a similar prevalence rate of TB and that the cases are likely to cluster at the district level (*24*). Getis’s G_i_^*^ statistics only assess positive spatial autocorrelation and are used to detect hot spots in the study area. A calculated value of G_i_^*^ >1.96 indicates that district *i* and its neighboring districts (within 10 km) have a TB prevalence rate that is statistically significantly different (higher) than other districts. District *i* is the center of the area with the higher TB prevalence rate, and is defined as a TB hot spot (*25*).

#### Model 1

To detect the difference of prevalence of TB among 18 districts, we constructed two 2-level statistical models (extra Poisson regression model) (*26*,*27*). The first level consisted of the following equation (details in the Technical Appendix ):

Case rate per unit of population = constantwhere case rate and constant are same as that in the first model. The second level (equation) of Model 1 examined the estimated coefficient for the constant and the statistical significance of the error term. If the error term is statistically significant, then it indicates that there are differences in the prevalence rate of TB at the district level (but does not identify which districts are different and the degree of difference).

#### Model 2

Our second statistical model assessed whether the migrant population was significantly associated with any differences between districts. Model 2 was also a 2-level statistical model, with the first level consisting of the following equation (details in the Technical Appendix ):

Case rate per unit population = constant + origin of case (zone) + age + gender

The second level (equation) of Model 2 examined the statistical significance of the error terms associated with the coefficients for both the constant and the zone of case origin. If the error term of the constant is statistically significant, then it indicates that there are differences in the prevalence rate of TB at the district level. If the error term for the zone of case origin is statistically significant, then it indicates that there are differences between resident and migrant populations.

## Results

Our study included 15,078 cases among the permanent residents and 7,948 cases among the migrant population diagnosed from January 1, 2004, through October 31, 2006, in Beijing. Of the migrant population during this time, 61.6% were male; 16.8% came from the western zone, 41.9% came from the middle zone, 40.5% came from the eastern zone, and 0.8% came from Tianjin and Shanghai.

Figure 2 shows the distribution of the permanent residents and migrant population among 18 districts from 2000 through 2006. During these 7 years, the permanent resident population and the migrant population were most dense in Xuanwu, Dongcheng, Chongwen, and Xicheng. The permanent resident population was stable and tended to decline, and the migrant population fluctuated and slightly increased. Figure 3 shows the prevalence rate of TB in the 2 populations. Except in Fengtai, the prevalence rate of TB in the permanent residents has tended to increase since 2004, which is in accordance with the prevalence in the migrant population.

When the 2 populations were compared among the 18 districts, a highly spatial cluster (z>1.96) of TB was shown at the district level (Table 1). The hot-spots among the migrant population persisted in Xuanwu, Chongwen, Xicheng, and Dongcheng from 2000 through 2006 (Appendix Table). For the permanent residents, however, global clusters were not detected, and just 1 hot spot, Miyun, was observed, in 2003 (Appendix Table). This implies that the epidemic of TB is dominated by the migrant population in Beijing.

**Table 1:** Moran’s *I* analysis on TB cases in the migrant population and permanent resident population, Beijing, 2000–2006*

Year	TB cases among Beijing permanent residents			TB cases among the migrant population	
	*I* statistic	z score		*I* statistic	z score
2000	–0.46	–1.51		0.17	0.94
2001	–0.43	–1.42		0.11	–0.25
2002	–0.27	–0.82		0.84	3.62†
2003	–0.14	–0.34		0.99	4.24†
2004	–0.34	–1.14		0.13	4.64†
2005	–0.24	–0.72		0.74	3.09†
2006	–0.36	–1.25		0.71	2.91†

*Moran’s *I* statistics with z score test value to detect the spatial distribution of tuberculosis (TB) in the 2 populations. A statistically significant (z score >1.96) estimate of *I* indicates that nearby districts (within 10 km) have a similar prevalence rate of TB and the cases are likely to cluster at the district level. †Statistically significant, p<0.05 as measured by z score >1.96.

The results from Model 1 indicate that statistically significant differences exist in the prevalence rates of TB at the district level (Table 2) The results from the first level of Model 2 show statistically significant difference in TB prevalence by district (the constant term), origin of cases (zone), gender, and age (Table 3). In the second level of this model, the error term associated with the origin (zone) of cases was significant, but the error term associated with district was not (Table 3).

**Table 2:** Results from Model 2: differences in prevalence rate of TB among the districts*

Parameter	Estimate	Standard error	χ^2^ value	p value
Level 1				
Constant†	–8.280	0.096	7378.941	<0.001
Level 2				
Error term associated with TB prevalence‡	0.150	0.056	7.229	0.0071

*TB, tuberculosis. †In this statistical model, the constant term represents the rate of TB by district. ‡If the error term is statistically significant, it indicates that there are differences in the prevalence rate of TB at the district level (but does not identify which districts are different and the degree of difference).

**Table 3:** Results from Model 3: differences in TB prevalence by district, origin of cases, gender and age of cases*

Parameter	Estimate	Standard error	χ^2^ value	p value
Level 1				
Constant	–9.823	0.117	6993.521	<0.001
Zone	0.795	0.144	30.397	<0.001
Age	0.948	0.035	737.1151	<0.001
Gender	0.501	0.031	269.385	<0.001
Level 2				
Error terms combined	0.200	0.069	8.330	<0.005
Error term associated with TB prevalence†	–0.067	0.068	0.965	0.3259
Error term associated with origin (zone) of cases‡	0.342	0.124	7.603	<0.005

*TB, tuberculosis. †If the error term is statistically significant, it indicates that there are differences in the prevalence rate of TB at the district level (but doesn’t identify which districts are different and the degree of difference). ‡If the error term is statistically significant, it indicates that there are differences in the prevalence rate of TB by origin (zone) of cases (but doesn’t identify which the degree of difference between origins).

## Discussion

Previous studies have indicated that the migrant population affects the prevalence of TB in Beijing (*9*–*13*). However, because of the limitation of the techniques used in the analysis of previous studies, the hot-spot distribution has not been displayed at the district level, and the potential association with the migrant population could not be analyzed quantitatively. In our study, we used GIS-based spatial analysis to elucidate the spatial distribution of TB and highlight the hot spot areas.

Population data showed that the 4 largest migrant populations live in the 4 suburban districts: Chaoyang, Haidian, Fengtai, and Changping (*8*,*9*,*19*–*23*). However, our study indicated that Chongwen, Xuanwu, Xicheng, and Dongcheng, the 4 central districts, were the hot spots of TB among the migrant population from 2000 through 2006. These central districts may be hot spots because the population densities of the migrants and the permanent residents in the 4 central districts are much higher than that in the suburban districts (Figure 2). In fact, ≈20% of the population crowd into the 4 center districts, which cover only 0.50% of the surface area (*8*,*9*,*19*–*23*), resulting in a high risk for transmission of TB from the migrant population to the permanent residents in these areas. Another explanation may be related to the education and the occupations of the migrant population. Data from Beijing economic census yearbook 2004 showed that most migrants were employed in commercial services in the 4 central districts (*28*), where they were likely to come into contact with the permanent residents. On the other hand, the migrants in the 4 suburban districts were mostly college students, construction workers, and technicians. These migrants usually resided in a given region and formed a special social group, which indicated that they would have less chance of coming into contact with persons outside their group. We are conducting further studies to investigate the effects of economic factors on TB prevalence, in particular, housing, life span, healthcare, gross domestic product, income, education, insurance, social welfare, and traffic.

The spatial analysis showed that TB is distributed randomly among permanent residents and that it tends to cluster in the migrant population; the correlation reached its peak in 2004 and declined in the next 2 years. The analytical result was consistent with the trend of TB in Beijing. During the previous 90 years, the incidence of TB had been controlled to 7 cases per 100,000 population and approached that of industrialized countries (*1*,*2*,*5*). With mass migrant populations pouring into Beijing, TB has reemerged in recent years. According to the report from the Beijing Municipal Health Bureau, annual new registered active cases held steady at ≈2,500 and slightly increased among the permanent residents from 2000 through 2006 (*1*) (Figure 3, panel A). To bring TB in the migrant population under control, some new control measures, such as free therapy (*29*) aimed at the migrant population, were carried out in Beijing, and the increase in disease was effectively halted. Our study also found that the migrant population made different contributions to the prevalence of TB. The national survey showed that TB prevalence in the western zone, with 451 active cases and 136 positive cases per 100,000 population, was ≈1.7× higher than the TB prevalence in the eastern zone and slightly higher than TB prevalence in the middle zone, with 438 active cases and 148 positive cases per 100,000 populations. The western zone was the area where TB was most serious, whereas the 3 municipalities (Beijing, Tianjin, and Shanghai) were the areas of lowest prevalence (*18*). A special study on socioeconomic factors attached to the national survey further disclosed that the differences in the epidemic of TB mainly originated from the economic inequalities among the 4 zones. The socioeconomic study pointed out that the income per person in 80% of families with patients was lower than that in the local population (*18*).

The results from our statistical models indicated that the effects of the migrant population on TB prevalence differ between districts. We can conclude that the migrant population is distributed asymmetrically in 18 districts and has different living conditions. Furthur investigation is needed to determine the underlying causes of TB in each district.

Our work was subject to several limitations. The first was that we performed a retrospective analysis. A prospective study would have been worthwhile for predicting the impact of the migrant population on the epidemic of TB. Businesspersons or travelers can become the transmitter of TB if they have been infected by *Mycobacterium tuberculosis.* The role of public transportation in the transmission of TB has been mentioned in many reports (*30*,*31*). However, the potential impact of businesspersons and travelers was not assessed in our study because we could not trace them and obtain reliable information about them. We have also noted that when the individual cases were aggregated to the district level, individual differences could be missed, and the effect of the individual on the disease was not taken into account. However, in the current study, we emphasized the effect of the whole migrant population on the epidemic of TB in each district. The delay or lag time between the initial outbreak of disease in the migrant population and the permanent residents was expected to affect the number of the permanent residents infected with *M. tuberculosis* (*32*) (data not shown).

In summary, our study confirmed that the migrant population contributed to the prevalence of TB and the differences among the 18 districts in Beijing. Our findings suggest that TB control measures should incorporate the migrant population, particularly persons from the western and middle zones. Our study also implied that further research is necessary on the correlation between TB and the economy.

## Supplementary Material

Appendix Table Tuberculosis hotspots in the permanent residents and the migrant population in Beijing, 2000-2006*

Technical Appendix Spatial Analysis of Tuberculosis Cases in Migrants and Permanent Residents, Beijing, 2000-2006
