# Supplementary material for: Spatial Analysis of Tuberculosis Cases in Migrants and Permanent Residents, Beijing, 2000–2006
Source: Emerg Infect Dis. 2008 Sep;14(9):1413–9. doi: 10.3201/1409.071543 (PMC2603090; doi:10.3201/1409.071543)
Supplement: Appendix Table — Tuberculosis hotspots in the permanent residents and the migrant population in Beijing, 2000-2006* [file 07-1543_appT-s1.pdf]

Appendix Table. Tuberculosis hotspots in the permanent residents and the migrant population in Beijing, 2000–2006*

Districts	Permanent residents							Migrant population						
	2000	2001	2002	2003	2004	2005	2006	2000	2001	2002	2003	2004	2005	2006
Dongcheng	0.26	0.53	0.32	–0.31	–0.59	–1.08	–0.89	**1.96**	1.60	**2.65**	**2.84**	**2.89**	**2.69**	**2.54**
Xicheng	0.94	1.08	0.71	0.43	0.10	–0.45	–0.19	**2.47**	**2.08**	**3.16**	**3.39**	**3.44**	**3.17**	**3.17**
Chongwen	0.26	0.53	0.32	–0.31	–0.59	–1.08	–0.89	**1.96**	1.60	**2.65**	**2.84**	**2.89**	**2.69**	**2.54**
Xuanwu	1.05	1.40	1.06	0.59	–0.09	–0.89	–0.86	**2.17**	1.84	**3.11**	**3.16**	**3.02**	**2.67**	**2.41**
Chaoyang	–0.04	0.42	–0.19	–1.00	–1.04	–1.27	–1.19	1.73	1.19	1.57	1.86	0.71	1.35	0.68
Fengtai	1.53	1.72	1.84	1.39	0.52	–0.30	–0.37	0.54	0.94	1.82	1.52	1.71	1.13	1.24
Shijingshan	–1.28	–1.24	–1.50	–1.42	–1.39	–1.29	–1.56	–0.20	–0.20	–0.55	–0.41	–0.90	–0.83	–0.92
Haidian	–0.24	0.15	0.49	0.57	1.04	1.37	0.45	–0.36	0.61	0.06	–0.24	–0.76	0.20	–0.93
Mentougou	–0.59	–0.73	–0.73	–0.67	–0.40	–0.48	–0.57	–0.73	–0.69	–0.49	–0.66	–0.60	–0.42	–0.77
Fangshan	0.13	–0.31	–0.24	0.11	0.27	0.25	–0.02	–0.15	–0.48	–0.47	–0.60	–0.05	–0.51	–0.51
Tongzhou	–0.26	–0.20	–0.14	0.06	–0.21	0.11	0.15	–0.83	–0.37	–0.56	0.38	–0.41	–0.59	–0.29
Shunyi	0.99	0.81	1.03	0.12	1.00	1.12	0.63	–0.75	–0.94	–0.71	–0.85	–0.65	0.03	–0.23
Changping	–0.24	0.15	0.49	0.57	1.04	1.37	0.45	–0.36	0.61	0.06	–0.24	–0.76	0.20	–0.93
Daxing	–0.56	–0.40	–1.01	–0.91	–0.15	–0.29	1.27	–0.19	0.00	–0.42	–0.69	–0.58	–0.88	–0.39
Pinggu	–0.70	–0.94	–1.00	–0.33	0.97	0.62	0.66	–0.91	–0.95	–0.79	–0.81	–0.81	–0.60	0.09
Huairou	–0.08	–0.04	0.34	–0.34	–0.78	–0.42	0.95	–1.19	–1.03	–0.85	–0.82	–0.93	–1.15	–0.86
Miyun	1.26	0.89	1.11	**2.57**	1.41	1.28	1.08	0.78	0.73	–0.58	–0.35	0.01	–0.31	–0.06
Yanqing	0.56	0.15	0.04	0.25	–0.64	0.24	–0.15	1.13	0.14	–0.20	0.32	0.66	0.28	1.31

*Getis's G_i^* statistics assess positive spatial autocorrelation. A calculated value of $G_i^* \geq 1.96$ indicates that district *i* and its neighboring districts (within 10 km) have a TB prevalence rate that is statistically significantly different (higher) than other districts. District *i* is the center of the area with the higher TB prevalence rate and is therefore defined as a TB "hot spot." **Boldface** indicates significant results (hot spots).
