# Supplementary material for: Spatial Analysis of Tuberculosis Cases in Migrants and Permanent Residents, Beijing, 2000–2006
Source: Emerg Infect Dis. 2008 Sep;14(9):1413–9. doi: 10.3201/1409.071543 (PMC2603090; doi:10.3201/1409.071543)
Supplement: Technical Appendix — Spatial Analysis of Tuberculosis Cases in Migrants and Permanent Residents, Beijing, 2000-2006 [file 07-1543_Techapp-s2.pdf]

Spatial Analysis of Tuberculosis Cases in Migrants and Permanent Residents, Beijing, 2000–2006

Technical Appendix

1. Detail of equation 1:

$$\text{case}_{ij} \sim \text{Poisson}(\pi_{ij})$$
$$\log(\pi_{ij}) = \text{offset}_{ij} + \beta_{0j}\text{cons}$$
$$\beta_{0j} = -8.280(0.096) + u_{0j}$$
$$\begin{bmatrix} u_{0j} \end{bmatrix} \sim \text{N}(0,\ \Omega_u)\ :\ \Omega_u = \begin{bmatrix} 0.150(0.056) \end{bmatrix}$$
$$\text{var}(\text{case}_{ij}|\pi_{ij}) = 8.050(0.285)\pi_{ij}$$

2. Detail of equation 2:

$$\text{case}_{ij} \sim \text{Poisson}(\pi_{ij})$$
$$\log(\pi_{ij}) = \text{offset}_{ij} + \beta_{0j}\text{cons} + \beta_{1j}\text{state}_{ij} + 0.948(0.035)\text{age} + 0.501(0.031)\text{gender}_{ij}$$
$$\beta_{0j} = -9.823(0.117) + u_{0j}$$
$$\beta_{1j} = 0.795(0.144) + u_{1j}$$
$$\begin{bmatrix} u_{0j} \\ u_{1j} \end{bmatrix} \sim \text{N}(0,\ \Omega_u)\ :\ \Omega_u = \begin{bmatrix} 0.200(0.069) & \\ -0.067(0.068) & 0.342(0.124) \end{bmatrix}$$
$$\text{var}(\text{case}_{ij}|\pi_{ij}) = 2.291(0.082)\pi_{ij}$$
